# A Rare Case of Mixed Neuroendocrine Tumor and Adenocarcinoma of the Pancreas

**DOI:** 10.1155/2016/3240569

**Published:** 2016-08-17

**Authors:** Sofia Xenaki, Konstantinos Lasithiotakis, Alexandros Andreou, Sofia Aggelaki, Maria Tzardi, Anna Daskalaki, George Chalkiadakis, Emmanuel Chrysos

**Affiliations:** ^1^Department of General Surgery, University General Hospital of Heraklion, Heraklion, 71110 Crete, Greece; ^2^Department of Medical Oncology, University General Hospital of Heraklion, Heraklion, 71110 Crete, Greece; ^3^Department of Pathology, University General Hospital of Heraklion, Heraklion, 71110 Crete, Greece

## Abstract

*Introduction.* Neuroendocrine carcinoma (NEC) of pancreas is a rare tumor with aggressive progression and poor prognosis. Its coexistence with adenocarcinoma poses significant clinical problems and has not been addressed in the literature.* Methods.* We describe a case of a 51-year-old male who underwent pancreatoduodenectomy due to pancreatic head tumor 1.5 × 1 × 1.4 cm. Histological examination of the specimen revealed a mixed neoplasm: (1) a well differentiated adenocarcinoma, neoplastic blasts of which are extended focally to the submucosa without invading the muscular layer, and (2) a low differentiated NEC consisting of solid clusters and pagetoid formations. All 18 lymph nodes of the specimen were free of neoplastic disease and the surgical margins of the specimen were tumor-free. No adjuvant treatment was administered and two months after the operation the patient developed liver metastasis. FNA cytology of the hepatic lesions revealed low grade carcinoma with neuroendocrine characteristics. Five lines of chemotherapy were administered: VP + CDDP, paclitaxel + ifosfamide + Mesna + CDDP, Folfox + Avastin, Folfiri + Avastin, and CAV. During his treatment he revealed PD and succumbed to his disease 13 months after the operation.* Conclusion.* Coexistence of NEC with adenocarcinoma of the pancreas is a very rare entity presenting significant challenges regarding its adjuvant treatment and the treatment of distant relapse.

## 1. Introduction

Neuroendocrine tumors are considered rare neoplasms with an annual incidence of 2.5–5 per 100.000 [1]. NETs are distributed in the gastrointestinal system and approximately 1/10 develop in pancreas. Their coexistence with pancreatic adenocarcinoma has been rarely described in the literature and the management of such mixed tumors is challenging mainly because of the significant differences regarding natural course and responsiveness to systemic therapy of each histological type [2]. Herein we report a rare case of a mixed malignant NET and adenocarcinoma of the pancreas and we discuss significant issues regarding various types of treatment used to control the disease.

## 2. Case Report

**Figure 1:**
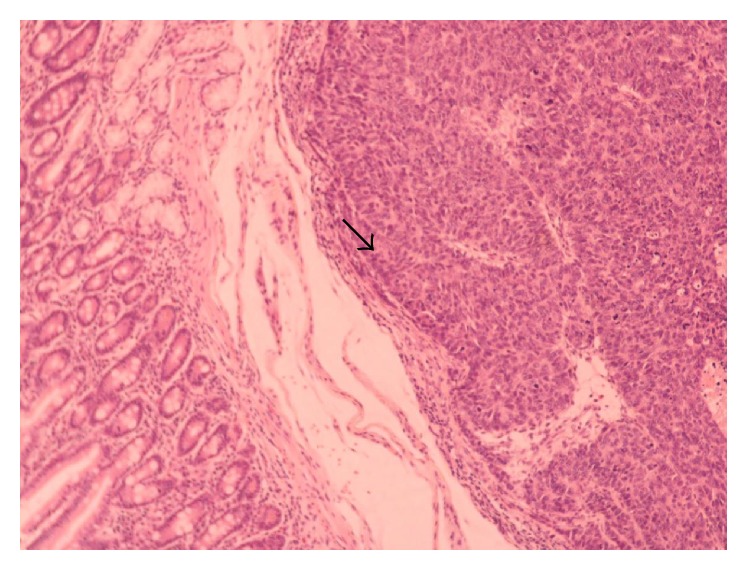
Adenocarcinoma of the pancreas.

**Figure 2:**
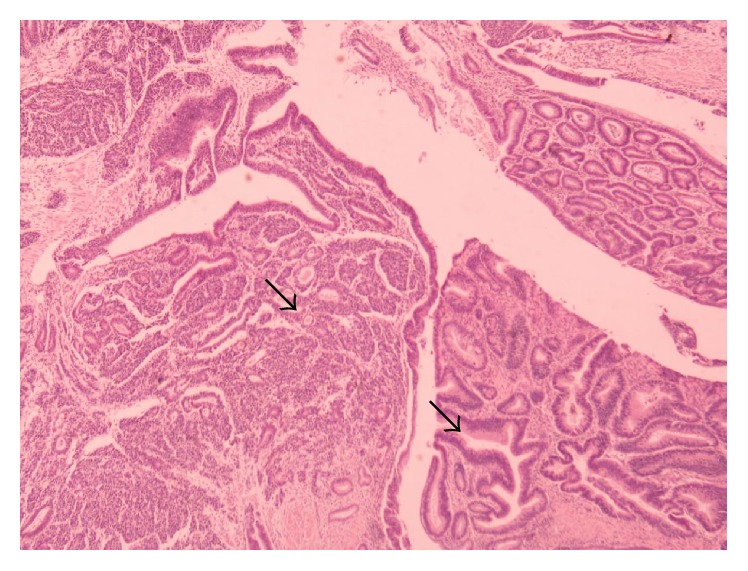
Mixed NET of the pancreas.

**Figure 3:**
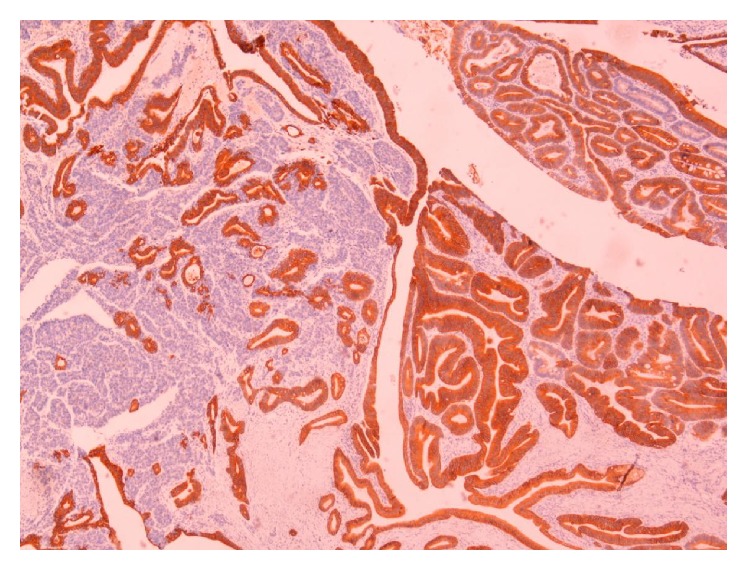
Mixed NET of the pancreas.

**Figure 4:**
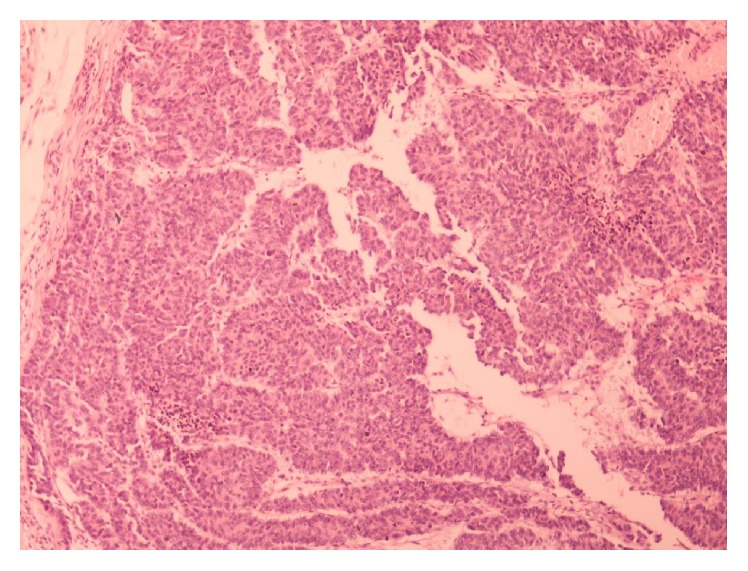
The tumor from the inside.

**Figure 5:**
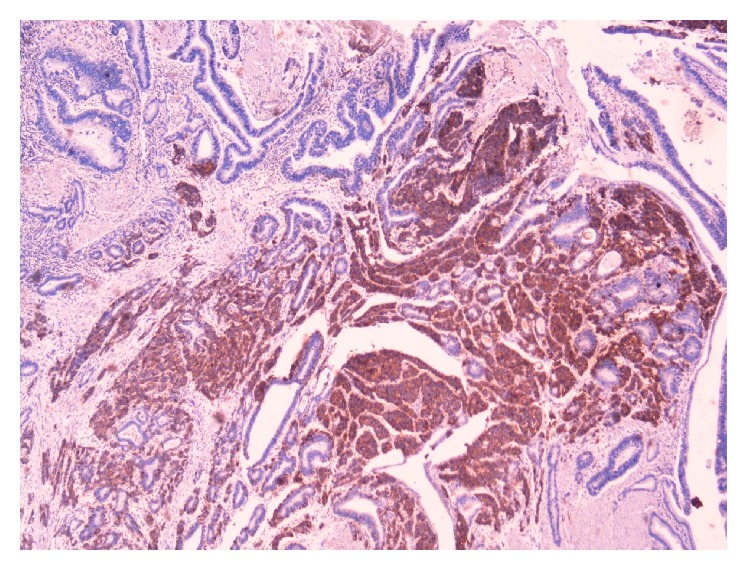
Cells stained with CD56.

A 51-year-old male was admitted to the hospital on April 2011 complaining of epigastric pain, jaundice, fever, chills, and billinuria. The lab results revealed increased levels of liver function test (AST, ALT, *γ*GT, and ALP). With the diagnosis of cholangitis the patient underwent ERCP which revealed biliary gallstones which were removed after sphincterotomy as well as abnormal tissue surrounding the papilla of Vater. Biopsy revealed an adenoma with moderate-to-high grade dysplasia where the possibility of infiltrative development could not be definitely excluded. Tumor markers (CEA, CA 19-9, and AFP) were within normal levels. On May 2011 a CT scan of the abdomen was conducted and revealed a compact homogenous tumor 1.5 × 1 × 1.4 cm invading the lumen of the 2nd section of the duodenum. Subsequently, the patient underwent pancreatoduodenectomy. Due to a superficial surgical site infection the hospitalization of the patient was prolonged until the 17th postoperative day. Histological examination of the specimen revealed a mixed neoplasm consisting of a part with histological characteristics of a well differentiated adenocarcinoma (Figure 1), neoplastic blasts of which were extending focally to the submucosa without invading the muscular layer (Figures 2 and 3). In the region of submucosa, there was low differentiation carcinoma consisting of solid clusters and pagetoid formations. The neoplastic cells of the former tumor were homogenous without obvious cytoplasm and they present a large number of mitoses (Figure 4). Immunohistochemical staining showed that the latter tumor was Cytokeratin 7(−), Cytokeratin 20(−), CEA(−), Cytokeratin MNF 116(+), CD56(+) (Figure 5), NSE(+), Synaptophysin(+), and Chromogranin(−). In contrast, the region of the well differentiated carcinoma was Cytokeratin 7(+), Cytokeratin 20(+), and CEA(+). Carcinomatous lymph embolus was obvious as well, but all 18 lymph nodes of the specimen were free of neoplastic disease and the surgical margins of the specimen were tumor-free.

Four months after the operation the patient developed liver metastasis. The CT scan showed 4 hypodense lesions in the liver segments V, VI, and VIII with respective maximum diameter of 13 mm, 2.5 cm, and 13 cm and hypodense lesion at the level of the hepatic vein ramification. FNA cytology of the hepatic lesions revealed low grade carcinoma with neuroendocrine characteristics and the patients received 1st-line chemotherapy treatment with VP and CDD: D1–D3 VP 100 mg/m^2^ and D2 CDDP 80 mg/m^2^. After 2 months, CT of the abdomen revealed progressive disease (PD) with 3.5 cm maximum lesion and received 2nd-line chemotherapy with paclitaxel, ifosfamide, Mesna, and CDDP (D1 paclitaxel 175 mg/m^2^, D1-D2 ifosfamide 2.5 g/m^2^, D1-D2 Mesna 1 gr/m^2^ before ifosfamide, 1 g/m^2^ 3 hrs after the ifosfamide dose, and 1 g/m^2^, 6 hrs later, and D1-D2 CDDP 40 mg/m^2^). Three months later due to PD (with the largest lesion being 3.7 cm) the patient received 3rd-line treatment with Folfox and Avastin (D1 Avastin 5 mg/kg, D1 LOHP 85 mg/kg, D1-D2 LV 200 mg/m^2^, D1-D2 STUp 400 mg/m^2^, and D1-D2 STUi 600 mg/m^2^). Two months later due to PD (with the largest lesion being 4.2 cm and appearance of new hepatic lesion as well) the patient received 4th-line chemotherapy with Folfiri and Avastin (D1 SFUbolus 400 mg/m^2^, D1 CPT 150 mg/m^2^, D1 LV 200 mg/m^2^, and D1-D2 SFUinf 600 mg/m^2^). Octreoscan revealed a region with moderate scintillation in the left hepatic lobe. Four months later the patient had PD (multiple hepatic lesions, with the largest one being 5.7 cm) and received 5th-line treatment with CAV: D1 Endoxan 1000 mg/m^2^, D1 Doxorubicin 40 mg/m^2^, and D1 Oncovin 1 mg/m^2^. In December 2012 the patient presented to the emergency department due to worsening general condition, malaise, weakness, and anemia. He was transfused with 3 blood units. A new CT scan of the abdomen revealed PD with multiple liver metastatic lesions with the largest one having 9.5 cm diameter. The patient experienced grade IV myelotoxicity without evidence of any response to chemotherapy and denied further treatment. He was discharged from the hospital and succumbed to his disease 13 months after the operation.

## 3. Discussion

The gastrointestinal NEC are considered to include those arising from the endocrine cells scattered among the epithelial cells (diffuse endocrine system) of the gastrointestinal tract and those derived from the endocrine cells that appear with the differentiation of adenoma and adenocarcinoma cells. Traditionally it was speculated that gastrointestinal endocrine tumors arose from (a) prior general adenocarcinomas, (b) prior carcinoid tumors, (c) nonneoplastic pluripotent stem cells, and (d) nonneoplastic immature endocrine cells. However, the structures of lesions and the results of genetic analysis have led to the current belief that mainly the lump-like growth of a highly proliferative, neoplastic endocrine cell clone appearing in the deep portion of the gland tubule of prior well and moderately differentiated, intramucosal, tubular adenocarcinomas results in the formation of gastrointestinal endocrine tumors via endocrine carcinomas [3].

Pancreatic neuroendocrine tumors are different from exocrine tumors of the pancreas (pancreatic adenocarcinoma), which account for about 95 percent of all pancreatic cancers [4]. Pancreatic neuroendocrine tumors are slow growing tumors that are fairly rare and are reported in two to four people per million annually worldwide [5, 6] and account for approximately 22–28 percent of all neuroendocrine tumors [7, 8]. The incidence of pancreatic neuroendocrine tumors appears to be rising, due in part to heightened awareness of the disease, improved diagnostic techniques, and an increased rate of incidental diagnoses during evaluations for other conditions [9, 10]. For patients with pancreatic neuroendocrine tumors that have metastasized, prognosis is poor, with a survival of only 1–3 years [11].

Concerning some more information according to the concurrent coexistence of adenocarcinoma and NET and the relationship between these two pathological entities it is rare to be found since these two entities appear together extremely rarely. In the literature very few cases have been described concerning NET with adenocarcinoma of the pancreas. Most published cases have described NET of the colon coexisting with adenocarcinoma of the sigmoid colon [12] or even ampulla of Vater with the sigmoid colon [13].

The natural history of islet cell and carcinoid tumors tends to be favorable as compared with pancreatic adenocarcinoma. For example, the median survival duration from the time of diagnosis for patients with nonfunctioning metastatic islet cell tumors approaches five years. The diagnosis of islet cell tumors is aided by the different abnormal biochemical profiles that they may present, which often leads to radiographic means to try and locate the tumor. It would be a mistake to generalize too much about attempts to locate these tumors. But, generally, dynamic CT scans with radiocontrast dye, octreotide scintigraphy, transabdominal ultrasound, and selective visceral angiography are all methods employed to elicit radiographic information about the cancer, depending on individual circumstance. Although they arise from similar cells, these different types of neuroendocrine cancers all behave somewhat differently. The standard treatments tend to be tumor type specific, but some general observations can be made. Immediate treatment of the symptomatic conditions created by the oversecretion of the hormone(s) may be appropriate (e.g., the use of H2-blockers, omeprazole, and even octreotide in gastrinomas). The treatment of choice for localized islet cell tumors is generally curative surgery. The treatment of metastatic islet cell cancer disease, depending on the tumor type, will often include chemotherapy involving such agents as streptozocin, everolimus, sunitinib, temozolomide, capecitabine, 5-FU, Doxorubicin, dacarbazine, and octreotide. Recently, promising studies of aggressive surgery benefiting select cases of metastatic neuroendocrine tumors have been published in the medical literature.

Therefore while there is a massive increase of metastatic pancreatic tumors and there is poor prognosis, we should be more aware as far as the treatment is concerned which should be chemotherapy or even palliative therapy when needed. We ought to offer a targeted and customized therapy to each patient.

## References

[1] La Rosa S., Marando A., Sessa F., Capella C. (2012). Mixed adenoneuroendocrine carcinomas (MANECs) of the gastrointestinal tract: an update. *Cancers*.

[2] Ehehalt F., Saeger H. D., Schmidt C. M., Grützmann R. (2009). Neuroendocrine tumors of the pancreas. *The Oncologist*.

[3] Noda Y., Watanabe H., Lwafuchi M. (1992). Carcinoids and endocrine cell micronests of the minor and major duodenal papillae. Their incidence and characteristics. *Cancer*.

[4] Mortelé K. J., Peters H. E., Odze R. D., Glickman J. N., Jajoo K., Banks P. A. (2009). An unusual mixed tumor of the pancreas: sonographic and MDCT features. *Journal of the Pancreas*.

[5] Ramage J. K., Davies A. H. G., Ardill J. (2005). Guidelines for the management of gastroenteropancreatic neuroendocrine (including carcinoid) tumours. *Gut*.

[6] Halfdanarson T. R., Rabe K. G., Rubin J., Petersen G. M. (2008). Pancreatic neuroendocrine tumors (PNETs): incidence, prognosis and recent trend toward improved survival. *Annals of Oncology*.

[7] Pape U.-F., Berndt U., Müller-Nordhorn J. (2008). Prognostic factors of long-term outcome in gastroenteropancreatic neuroendocrine tumours. *Endocrine-Related Cancer*.

[8] Ter-Minassian M., Frauenhoffer C. S., Hooshmand S. M. Prospective analysis of clinical outcomes and prognostic factors in patients with Neuroendocrine tumors (NETs).

[9] Cheema A., Weber J., Kvols L., Strosberg J. Incidental diagnosis of pancreatic neuroendocrine tumors.

[10] Öberg K. E. (2010). Gastrointestinal neuroendocrine tumors. *Annals of Oncology*.

[11] Yao J. C., Eisner M. P., Leary C. (2007). Population-based study of islet cell carcinoma. *Annals of Surgical Oncology*.

[12] Katalinic D., Santek F., Juretic A., Skegro D., Plestina S. (2014). Gastroenteropancreatic neuroendocrine tumour arising in Meckel's diverticulum coexisting with colon adenocarcinoma. *World Journal of Surgical Oncology*.

[13] Cokmert S., Demir L., Akder Sari A. (2013). Synchronous appearance of a high-grade neuroendocrine carcinoma of the ampulla vater and sigmoid colon adenocarcinoma. *Case Reports in Oncological Medicine*.

